# Levofloxacin-associated tendon disorders: Sex-, age-, and route-specific disproportionality analysis using FAERS and JADER

**DOI:** 10.1371/journal.pone.0358427

**Published:** 2026-09-11

**Authors:** Toshihisa Nakashima, Yoshihiro Noguchi, Rikuto Masuda, Tomoaki Yoshimura

**Affiliations:** 1 Laboratory of Clinical Pharmacy, Gifu Pharmaceutical University, Gifu, Japan; 2 Department of Pharmacy, National Cancer Center Hospital, Tokyo, Japan; 3 Office of Infection Control and Prevention, National Cancer Center Hospital, Tokyo, Japan; 4 Endowed Course of Social Community Pharmacy, Gifu Pharmaceutical University, Gifu, Japan; Universiti Malaya, MALAYSIA

## Abstract

This study evaluated the association between individual fluoroquinolones and tendon disorders in Japan using two spontaneous reporting systems: the Food and Drug Administration Adverse Event Reporting System (FAERS) and the Japanese Adverse Drug Event Report (JADER). Five agents—ciprofloxacin, levofloxacin, moxifloxacin, norfloxacin, and ofloxacin—were analyzed for “Muscle, tendon and ligament injuries” and “Tendon disorders.” All fluoroquinolones showed positive signals in the FAERS, whereas only levofloxacin demonstrated consistent and robust associations in the Japanese subset of the FAERS and the JADER. In the JADER, levofloxacin exhibited strong signals for muscle, tendon, and ligament injuries (reporting odds ratio [ROR] 30.6, information component [IC] 4.5) and tendon disorders (ROR 134.2, IC 5.8). Stratified analysis revealed markedly stronger signals in males, particularly those aged ≥70 years (relative ROR 12.21 and 25.67; IC delta 3.18 and 2.85, respectively). Oral levofloxacin was associated with higher reporting odds than intravenous administration. Time-to-onset analysis indicated that most tendon-related adverse events occurred within 14 days after drug initiation. Levofloxacin exhibited the most consistent and robust association with tendon disorders, especially in males aged ≥70 years in Japan. Therefore, clinicians in Japan should exercise caution when prescribing levofloxacin to older male patients.

## Introduction

Fluoroquinolones are clinically important antibiotics with high oral bioavailability, extensive tissue distribution, and broad-spectrum antimicrobial activity [1]. Despite their widespread clinical use, fluoroquinolones are associated with a range of adverse effects, some of which are rare but potentially serious. These adverse events include peripheral neuropathy, aortic aneurysm or dissection, dysglycemia (hypoglycemia and hyperglycemia), QT interval prolongation with torsades de pointes, *Clostridioides difficile* infection, neuropsychiatric adverse effects, tendinopathy, and tendon rupture [2–8].

Fluoroquinolone-associated tendinopathy and tendon rupture have been reported across several study designs, including systematic reviews and population-based evaluations [2,9]. Disproportionate reporting of tendinopathy and tendon rupture has also been observed in large pharmacovigilance datasets such as the US Food and Drug Administration Adverse Event Reporting System (FAERS) and VigiBase, with FAERS-based analyses identifying associations with ciprofloxacin, levofloxacin, and moxifloxacin [10,11]. Contrastingly, evidence from Asia, particularly Japan, remains limited and fragmented, with most data derived from case reports and small-scale or single-center studies. In a single-center analysis in Japan, Hori et al. reported 14 tendon disorder events among 17,147 patients exposed to fluoroquinolones (levofloxacin, n = 9; moxifloxacin, n = 2; tosufloxacin, n = 2; ofloxacin, n = 1) and a higher risk of tendon disorder events with fluoroquinolones than with cephalosporins [12]. However, given the modest event counts and single-center setting, conclusions about agent-specific risks in Japanese populations should be regarded as preliminary and interpreted with caution. Accordingly, the risk of tendinopathy or tendon rupture associated with individual fluoroquinolones remains unclear in Japan.

The mechanisms underlying fluoroquinolone-associated tendon disorders have not yet been fully elucidated. However, several hypotheses have been proposed. First, in extracellular matrix degradation, fluoroquinolones upregulate matrix metalloproteinases (MMPs) such as MMP-2 and MMP-3, thereby enhancing the degradation of type I collagen [13,14]. Second, in impaired repair dynamics, fluoroquinolones inhibit the speed and migration of tenocytes by downregulating focal adhesion kinase phosphorylation [14,15]. Third, oxidative stress and mitochondrial dysfunction have been implicated. Additionally, experimental models have demonstrated mechanisms such as increased reactive oxygen species, oxidation of type I collagen, and caspase-mediated apoptosis; N-acetylcysteine attenuates these effects [16].

In this study, we evaluated the association between individual fluoroquinolones and tendon disorders in Japan and assessed the differences by age, sex, and route of administration. Disproportionality analyses were conducted using spontaneous reports from the Japanese Adverse Drug Event Report (JADER) database and FAERS.

## Materials and methods

All the analyses were conducted in accordance with the ethical principles of the Declaration of Helsinki for medical research. As the data were anonymized and all analyses were descriptive, informed consent from patients was not required, and ethical review by an institutional review board was deemed unnecessary. This study was conducted according to The Reporting of A Disproportionality Analysis for Drug Safety Signal Detection Using Individual Case Safety Reports in Pharmacovigilance guidelines [17,18].

### Data source

We used case data from two pharmacovigilance resources: the JADER database (September 2024 release version) and JAPIC AERS, a curated distribution of the FAERS, covering Q4 1997–Q2 2024. JADER, maintained by the Pharmaceuticals and Medical Devices Agency, is publicly accessible (in Japanese only) at https://www.info.pmda.go.jp/fukusayoudb/CsvDownload.jsp (accessed on October 6, 2025). It is distributed as four comma-separated value (CSV) files: DEMO.csv (patient demographics), DRUG.csv (drug information), HIST.csv (medical history), and REAC.csv (adverse event information). FAERS quarterly files beginning with Q4 2004 are available for download on the FDA website. Earlier data (1997–2003) were archived by the US National Technical Information Service. These files are included in the JAPIC AERS database. The FAERS contains duplicate data, and the DRUG data are not standardized in terms of product name notation, ingredient name, specification, and dosage form. Some were misspelled. Therefore, when using the FAERS data for research purposes, it is necessary to clean the data and remove duplicates. The JAPIC AERS data have already been clean and deduplicated; the data can be referenced by the drug or brand name to identify the ingredient name. In this study, we excluded records in which involvement of drugs in adverse drug reactions was listed as ‘concomitant drug’ or was missing in JADER, as well as records in which the role_code was ‘concomitant’ or missing in JAPIC AERS. Patients with missing age were excluded from the analysis. Sex was restricted to male and female. In FAERS, we constructed a Japan-restricted FAERS subset (FAERS-Japan), retaining records with JP in OCCR_COUNTRY, or when OCCR_COUNTRY was missing, JPN in COUNTRY_CODE. Institutional review board permission was not required because this was an observational study using data from an open-access database containing anonymized patient information. Informed consent was not required because it was not possible to identify specific individuals among patients.

### Definitions of suspected drugs and adverse events

Fluoroquinolones (ciprofloxacin, levofloxacin, moxifloxacin, norfloxacin, and ofloxacin) were selected for the investigation. The five target fluoroquinolones were identified using generic names and their salt forms, as listed in S1 Table. Because JAPIC AERS standardizes product and brand names to ingredient names during database curation, additional brand-name searching was not performed. WHO Drug Dictionary or RxNorm was not used for additional drug-name standardization. The preferred terms from the Medical Terminology for Regulatory Activities/Japanese version were used to identify adverse events. We evaluated the target adverse events using high-level terms (HLTs) [19]. The preferred terms included in the HLTs for “muscle, tendon, and ligament injuries” and “tendon disorders” were included as target adverse events in this study. The complete list of Preferred Terms included in each target HLT is shown in S2 Table.

### Signal detection

In this study, disproportionality signals were assessed for drug-induced muscle, tendon, and ligament injuries and tendon disorders, using the reporting odds ratio (ROR) and relative ROR (rROR) as the frequentist statistical approach and the information component (IC) and IC delta (IC_Δ_) as the Bayesian confidence propagation neural network (BCPNN) method [20]. The ROR was calculated using a 2 × 2 contingency table (Table 1), according to Equations (1) and (2). In the present study, the analyses were stratified according to age and sex. Signal detection in sex or age was defined using the rROR reported by the European Medicines Agency and derived from a 4 × 2 contingency table (Table 2) and Equations (3)–(6) [20]. The ROR, rROR, and their 95% confidence intervals (CIs) were estimated. We applied a lower bound of the 95% CI (ROR_025_, rROR_025_) > 1 [21]. The ROR was not calculated when no reports were available. IC and IC_Δ_ were calculated using Tables 1 and 2 and Equations (7)–(12). Their 95% credible intervals (95% CrIs) were estimated. The 95% CrI of the IC_Δ_ was calculated using the same IC method. IC_025_ and IC_975_ are the lower and upper limits of 95% CrI, respectively. IC- and IC_Δ_-based signal was defined as the lower bound of the interval, the 2.5th percentile of CrI (IC_025_, IC_Δ025_) > 0 [22]. In this study, the criteria for signal detection were considered significant when identified using both the frequentist statistical approach and BCPNN methods.

**Table 1:** The 2 × 2 contingency table for signal detection.

	Target AEs	Other AEs	Total
Target drug	*N*_11_	*N*_10_	*N*_1+_
Other drugs	*N*_01_	*N*_00_	*N*_0+_
Total	*N* _+ 1_	*N* _+ 0_	*N*_++_

*N*: the number of reports; AE: adverse event.

**Table 2:** The 4 × 2 contingency table for two group comparisons.

	Target AEs	Other AEs	Total
Group_A_			
Target drug	*N*_A11_	*N*_A10_	*N*_A1+_
Other drugs	*N*_A01_	*N*_A00_	*N*_A0+_
Group_not A_			
Target drug	*N*_not A11_	*N*_not A10_	*N*_not A1+_
Other drugs	*N*_not A01_	*N*_not A00_	*N*_not A0+_
Total	*N* _+ 1_	*N* _+ 0_	*N*_++_


$$\begin{array}{c} \text{ROR }=\;\frac{\frac{N_{\text{11}}}{N_{\text{10}}}}{\frac{N_{\text{01}}}{N_{\text{00}}}} \end{array}$$ (1)

$$\begin{array}{c} \text{ROR }(\text{95}\%\text{ CI})\;=\;e^{\text{ln}(\text{ROR})\pm \text{1}.\text{96}\sqrt{\frac{\text{1}}{N_{\text{11}}}\;+\;\frac{\text{1}}{N_{\text{10}}}\;+\;\frac{\text{1}}{N_{\text{01}}}\;+\;\frac{\text{1}}{N_{\text{00}}}}} \end{array}$$ (2)

$$\begin{array}{c} {\text{Relative ROR}}_{A/not\;A}=\;\frac{{\text{ROR}}_{A}}{{\text{ROR}}_{not\;A}} \end{array}$$ (3)

$$\begin{array}{c} S_{{\text{Relative ROR}}_{A/not\;A}}\;=\;\sqrt{S_{{\text{ROR}}_{A}}^{\text{2}}+S_{{\text{ROR}}_{not\;A}}^{\text{2}}} \end{array}$$ (4)

$$\begin{array}{c} {\text{Relative ROR}}_{A/not\;A\text{0}\text{2}\text{5}}\;=\;\frac{{\text{Relative ROR}}_{A/not\;A}}{e^{{\text{1}.\text{9}\text{6}}_{S_{{\text{Relative ROR}}_{A/not\;A}}}}} \end{array}$$ (5)

$$\begin{array}{c} {\text{Relative ROR}}_{A/not\;A\text{9}\text{7}\text{5}}\;=\;{\text{Relative ROR}}_{A/not\;A}\;\times e^{{\text{1}.\text{9}\text{6}}_{S_{{\text{Relative ROR}}_{A/not\;A}}}} \end{array}$$ (6)

$$\begin{array}{c} \text{OE }=\;\frac{\text{O}(\text{Observed})}{\text{E}(\text{Expected})}\;=\;\frac{N_{\text{1}\text{1}}}{N_{+\text{1}}N_{\text{1}+}/N_{++}} \end{array}$$ (7)

$$\begin{array}{c} \text{IC }\approx \;{\text{log}}_{\text{2}}\frac{\text{O}+\text{0}.\text{5}}{\text{E}+\text{0}.\text{5}} \end{array}$$ (8)

$$\begin{array}{c} {\text{IC}}_{\text{0}\text{2}\text{5}}\;={\text{log}}_{\text{2}}\frac{\text{O}+\text{0}.\text{5}}{\text{E}+\text{0}.\text{5}}-\text{3}.\text{3}\times ({\text{O}+\text{0}.\text{5})}^{-\frac{\text{1}}{\text{2}}}-\text{2}\times (\text{O}+\text{0}.\text{5}{)}^{-\text{3}/\text{2}} \end{array}$$ (9)

$$\begin{array}{c} {\text{IC}}_{\text{9}\text{7}\text{5}}={\text{log}}_{\text{2}}\frac{\text{O}+\text{0}.\text{5}}{\text{E}+\text{0}.\text{5}}+\text{2}.\text{4}\times ({\text{O}+\text{0}.\text{5})}^{-\frac{\text{1}}{\text{2}}}-\text{0}.\text{5}\times (\text{O}+\text{0}.\text{5}{)}^{-\text{3}/\text{2}} \end{array}$$ (10)

$$\begin{array}{c} \text{O}{\text{E}}_{\Delta }=\frac{\text{O}{\text{E}}_{A}}{\text{O}{\text{E}}_{not\;A}}=\frac{{\text{O}}_{A}}{{\text{E}}_{A}{\text{O}}_{not\;A}/E_{not\;A}}=\frac{{\text{O}}_{A}}{{\text{E}}^{*}} \end{array}$$ (11)

$$\begin{array}{c} {\text{IC}}_{\Delta }\approx {\text{log}}_{\text{2}}\frac{{\text{O}}_{A}+\text{0}.\text{5}}{{\text{E}}^{*}+\text{0}.\text{5}} \end{array}$$ (12)

### Statistical analysis

Continuous variables were compared using Welch’s test. Statistical significance was defined as a two-tailed P-value < 0.05. All statistical analyses were performed using the R software (version 4.4.2; R Foundation for Statistical Computing, Vienna, Austria).

### Results

#### Patient characteristics in FAERS.

After excluding reports that used subjective terms such as “youth” and “elderly,” as well as those lacking information on sex and age, 10,855,976 patient reports (all cases) were obtained in the FAERS dataset. Ciprofloxacin, levofloxacin, moxifloxacin, norfloxacin, and ofloxacin were used in 44,814, 39,375, 17,415, 2,115, and 4,532 cases, respectively. Tables 3 and 4 show the characteristics of the patients using these fluoroquinolones and their distribution according to sex and age.

**Table 3:** Characteristics of patients with muscle, tendon and ligament injuries using fluoroquinolones and their distribution by sex and age in FAERS.

	Ciprofloxacin				Levofloxacin				Moxifloxacin				Norfloxacin				Ofloxacin			
	Male		Female		Male		Female		Male		Female		Male		Female		Male		Female	
	*N*_11_	*N*_1+_	*N*_11_	*N*_1+_	*N*_11_	*N*_1+_	*N*_11_	*N*_1+_	*N*_11_	*N*_1+_	*N*_11_	*N*_1+_	*N*_11_	*N*_1+_	*N*_11_	*N*_1+_	*N*_11_	*N*_1+_	*N*_11_	*N*_1+_
Total	2088	19780	2299	25034	2526	17176	3209	22199	317	7003	423	10412	48	711	67	1404	150	2250	112	2282
< 20	27	896	36	1024	13	382	28	533	0	214	3	273	0	15	2	49	4	125	0	127
20–29	179	1355	138	1833	98	800	85	1082	11	429	32	766	2	44	1	103	4	114	4	137
30–39	361	2138	288	2661	182	1382	275	2125	44	791	70	1265	9	89	6	195	17	174	12	245
40–49	368	2390	473	3423	381	2016	561	3071	73	982	92	1666	8	93	15	161	27	239	14	191
50–59	368	3236	519	4306	444	2713	835	4138	76	1185	137	2043	5	116	19	161	16	337	30	233
60–69	392	3969	453	4732	548	3691	722	4323	70	1290	53	1977	9	159	15	292	24	463	34	432
70–79	252	3533	268	3913	550	3774	471	4024	22	1314	25	1390	5	104	8	245	32	444	7	438
80–89	136	2010	105	2560	279	2087	216	2288	15	678	10	846	10	87	1	159	26	302	10	351
≥ 90	5	253	19	582	31	331	16	595	6	120	1	186	0	4	0	39	0	52	1	128

*N*_11_: the number of target-drug-induced muscle, tendon and ligament injuries; *N*_1+_: the number of all target-drug-induced adverse events.

**Table 4:** Characteristics of patients with tendon disorders using fluoroquinolones and their distribution by sex and age in FAERS.

	Ciprofloxacin				Levofloxacin				Moxifloxacin				Norfloxacin				Ofloxacin			
	Male		Female		Male		Female		Male		Female		Male		Female		Male		Female	
	*N*_11_	*N*_1+_	*N*_11_	*N*_1+_	*N*_11_	*N*_1+_	*N*_11_	*N*_1+_	*N*_11_	*N*_1+_	*N*_11_	*N*_1+_	*N*_11_	*N*_1+_	*N*_11_	*N*_1+_	*N*_11_	*N*_1+_	*N*_11_	*N*_1+_
Total	2710	19780	2909	25034	2863	17176	3661	22199	379	7003	427	10412	48	711	74	1404	191	2250	133	2282
< 20	37	896	46	1024	16	382	35	533	0	214	3	273	0	15	2	49	3	125	0	127
20–29	246	1355	202	1833	122	800	104	1082	18	429	36	766	2	44	1	103	9	114	5	137
30–39	557	2138	445	2661	244	1382	355	2125	62	791	84	1265	10	89	11	195	38	174	17	245
40–49	482	2390	628	3423	435	2016	652	3071	92	982	80	1666	8	93	14	161	30	239	17	191
50–59	446	3236	643	4306	489	2713	976	4138	89	1185	122	2043	5	116	19	161	22	337	34	233
60–69	499	3969	508	4732	604	3691	799	4323	73	1290	55	1977	10	159	15	292	35	463	41	432
70–79	289	3533	291	3913	580	3774	487	4024	25	1314	37	1390	3	104	10	245	33	444	10	438
80–89	149	2010	123	2560	343	2087	229	2288	16	678	9	846	10	87	2	159	21	302	8	351
≥ 90	5	253	23	582	30	331	24	595	4	120	1	186	0	4	0	39	0	52	1	128

*N*_11_: the number of target-drug-induced tendon disorders; *N*_1+_: the number of all target-drug-induced adverse events.

### Fluoroquinolone-associated muscle, tendon, and ligament injuries and tendon disorders in FAERS

Using the three disproportionality algorithms, we summarized the signals for muscle, tendon, and ligament injuries and tendon disorders in the overall FAERS dataset (Tables 5 and 6). Five fluoroquinolones (levofloxacin, ciprofloxacin, moxifloxacin, norfloxacin, and ofloxacin) exhibited positive disproportionality signals for both outcomes. Levofloxacin exhibited positive disproportionality signals for muscle, tendon, and ligament injuries (*N*_11_, 5,735; ROR, 38.7; 95% CI, 37.6–39.9; IC, 4.8; 95% CrI, 4.8–4.9) and tendon disorders (*N*_11_, 6,524; ROR, 82.6; 95% CI, 80.3–85.1; IC, 5.7; 95% CrI, 5.7–5.8) with the highest positive signal values. Ciprofloxacin also exhibited positive disproportionality signals for muscle, tendon, and ligament injuries (*N*_11_, 4,387; ROR, 23.9; 95% CI, 23.2–24.7; IC, 4.3; 95% CrI, 4.2–4.3) and tendon disorders (*N*_11_, 5,619; ROR, 57.6; 95% CI, 55.9–59.4; IC, 5.3; 95% CrI, 5.3–5.4). In subsequent analyses, we restricted the FAERS data to reports submitted from Japan (FAERS-Japan) and evaluated the same endpoints (Tables 5 and 6). In FAERS-Japan, levofloxacin exhibited positive disproportionality signals for muscle, tendon, and ligament injuries (*N*_11_, 24; ROR, 10.2; 95% CI, 6.7–15.4; IC, 3.0; 95% CrI, 2.3–3.5) and tendon disorders (*N*_11_, 21; ROR, 10.2; 95% CI, 6.7–15.4; IC, 3.8; 95% CrI, 3.1–4.3). There were no reports of muscle, tendon, or ligament injuries or tendon disorders involving norfloxacin or ofloxacin, and no positive disproportionality signals were detected for ciprofloxacin or moxifloxacin.

**Table 5:** Signal detection for fluoroquinolone-Induced muscle, tendon and ligament Injuries in FAERS.

	*N*_11_	ROR (95% CI)	IC (95% CrI)
All			
Ciprofloxacin	4387	23.9 (23.2–24.7)	4.3 (4.2–4.3)
Levofloxacin	5735	38.7 (37.6–39.9)	4.8 (4.8–4.9)
Moxifloxacin	740	9.1 (8.4–9.8)	3.1 (2.9–3.1)
Norfloxacin	115	11.7 (9.7–14.1)	3.4 (3.1–3.6)
Ofloxacin	262	12.5 (11.0–14.2)	3.5 (3.3–3.6)
Japan			
Ciprofloxacin	2	6.7 (1.6–27.1)	1.6 (−0.9–3.0)
Levofloxacin	24	10.2 (6.7–15.4)	3.0 (2.3–3.5)
Moxifloxacin	1	3.0 (0.4–22.1)	0.8 (−2.9–2.5)

*N*_11_: the number of target-drug-induced muscle, tendon and ligament injuries; ROR: reporting odds ratio; CI: confidence interval; IC: information component; CrI: credible interval.

**Table 6:** Signal detection for fluoroquinolone-Induced tendon disorders in FAERS.

	*N*_11_	ROR (95% CI)	IC (95% CrI)
All			
Ciprofloxacin	5619	57.6 (55.9–59.4)	5.3 (5.3–5.4)
Levofloxacin	6524	82.6 (80.3–85.1)	5.7 (5.7–5.8)
Moxifloxacin	806	16.5 (15.4–17.8)	3.9 (3.8–4.0)
Norfloxacin	122	20.4 (17.0–24.6)	4.1 (3.8–4.3)
Ofloxacin	324	25.9 (23.1–29.0)	4.5 (4.3–4.6)
Japan			
Ciprofloxacin	1	8.2 (1.1–58.9)	1.2 (−2.5–2.9)
Levofloxacin	21	10.2 (6.7–15.4)	3.8 (3.1–4.3)
Moxifloxacin	1	7.6 (1.0–54.9)	1.2 (−2.5–2.9)

*N*_11_: the number of target-drug-induced tendon disorders; ROR: reporting odds ratio; CI: confidence interval; IC: information component; CrI: credible interval.

### Age and sex stratification of signals in FAERS compared with JADER

As in FAERS, disproportionality analyses were performed in JADER; however, there were no reports of muscle, tendon, or ligament injuries or tendon disorders with moxifloxacin or ofloxacin, and *N*_11_ was low for norfloxacin (1 and 1). For ciprofloxacin, a weak positive disproportionality signal was exhibited for tendon disorders (*N*_11_, 4; ROR, 7.6; 95% CI, 2.8–20.4; IC, 2.1; 95% CrI, 0.3–3.2), whereas no signal was detected for muscle, tendon and ligament injuries (*N*_11_, 3; ROR, 2.0; 95% CI, 0.6–6.3; IC, 0.8; 95% CrI, −1.2–2.0). For levofloxacin, disproportionality signals for muscle, tendon, and ligament injuries and tendon disorders were compared between the overall FAERS and JADER datasets (Tables 7 and 8). In JADER, levofloxacin exhibited positive disproportionality signals for muscle, tendon, and ligament injuries (*N*_11_, 247; ROR, 30.6; 95% CI, 26.6–35.2; IC, 4.5; 95% CrI, 4.3–4.6) and tendon disorders (*N*_11_, 245; ROR, 134.2; 95% CI, 112.5–160.2; IC, 5.8; 95% CrI, 5.6–6.0). For levofloxacin, sex-stratified analyses detected positive disproportionality signals for muscle, tendon, and ligament injuries and tendon disorders in both males and females in the FAERS and JADER databases. In both databases, the signals were significantly stronger in males than in females. In age-stratified analyses of JADER, there were no reports of muscle, tendon, or ligament injuries or tendon disorders among males aged <20 years, and no reports among females aged <30 years or ≥90 years. In JADER, positive disproportionality signals were identified for muscle, tendon, and ligament injuries across all strata except among females aged ≥70 years. Contrastingly, tendon disorders showed positive disproportionality signals in both males and females across both age groups (≥ 70 and < 70 years) (Table 9). In the ≥ 70 years group, a significantly stronger disproportionality signal was detected in male than in female both muscle, tendon and ligament injuries (rROR, 12.21; 95% CI, 4.85–30.72; IC_Δ_, 3.18; 95% CrI, 2.81–3.45 [reference: female]) and tendon disorders (rROR, 25.67; 95% CI, 8.37–78.72; IC_Δ_, 2.85; 95% CrI, 2.48–3.11 [reference: female]).

**Table 7:** Comparison of FAERS and JADER signals for levofloxacin-associated muscle, tendon and ligament Injuries by sex and age.

	FAERS					JADER				
	*N*_11_	ROR (95% CI)	IC (95% CrI)	rROR (95%CI)	IC_Δ_ (95%CrI)	*N*_11_	ROR (95% CI)	IC (95% CrI)	rROR (95%CI)	IC_Δ_ (95%CrI)
ALL	5735	38.7 (37.6–39.9)	4.8 (4.8–4.9)			247	30.6 (26.6–35.2)	4.5 (4.3–4.6)		
Male	2526	42.5 (40.6–44.4)	4.9 (4.9–5.0)	1.16 (1.09–1.23)	0.16 (0.10–21)	178	38.7 (32.6–45.8)	4.7 (4.5–4.9)	1.91 (1.40–2.59)	0.74 (0.49–0.92)
Female	3209	36.5 (35.1–37.9)	4.8 (4.7–4.8)			69	20.2 (15.6–26.1)	3.9 (3.5–4.2)		
Male										
< 20	13	13.5 (7.7–23.7)	3.1 (2.2–3.8)			0				
20–29	98	36.7 (29.4–45.9)	4.6 (4.3–4.9)			0				
30–39	182	31.8 (27.0–37.5)	4.5 (4.3–4.7)			11	38.1 (18.2–79.5)	3.6 (2.5–4.2)		
40–49	381	43.3 (38.5–48.8)	4.8 (4.7–5.0)			24	57.7 (34.5–96.5)	4.3 (3.6–4.8)		
50–59	444	40.5 (36.4–45.1)	4.8 (4.7–5.0)			7	20.4 (9.2–45.3)	3.0 (1.7–3.9)		
60–69	548	40.9 (37.2–45.0)	4.9 (4.7–5.0)			57	72.7 (52.8–100.2)	5.0 (4.6–5.3)		
70–79	550	50.8 (46.0–56.0)	5.1 (4.9–5.2)			63	54.5 (40.8–72.9)	4.9 (4.4–5.2)		
80–89	279	53.2 (46.3–61.2)	5.1 (4.9–5.2)			15	14.7 (8.5–25.3)	3.2 (2.3–3.8)		
≥ 90	31	40.6 (26.9–61.1)	4.3 (3.7–4.7)			1	9.3 (1.1–72.8)	1.2 (-2.5)		
Female										
< 20	28	49.4 (33.3–73.1)	4.6 (4.0–5.0)			0				
20–29	85	44.1 (35.0–55.5)	4.9 (4.5–5.2)			0				
30–39	275	48.4 (42.3–55.3)	5.1 (4.9–5.2)			18	71.9 (38.1–135.4)	4.2 (3.4–4.7)		
40–49	561	33.1 (30.1–36.4)	4.6 (4.4–4.7)			2	10.0 (2.3–42.3)	1.8 (-0.7–3.2)		
50–59	835	41.4 (38.3–44.9)	4.8 (4.7–4.9)			27	62.1 (39.1–98.5)	4.5 (3.9–5.0)		
60–69	722	36.8 (33.9–40.0)	4.7 (4.6–4.8)			17	34.0 (20.0–57.7)	3.9 (3.1–4.5)		
70–79	471	29.9 (27.1–33.1)	4.5 (4.4–4.6)			3	4.0 (1.2–12.8)	1.4 (-0.5–2.6)		
80–89	216	31.2 (26.9–36.2)	4.5 (4.3–4.7)			2	2.3 (0.5–9.7)	0.8 (-1.7–2.2)		
≥ 90	16	10.4 (6.2–17.4)	2.9 (2.0–3.5)			0				

*N*_11_: the number of target-drug-induced target adverse event; ROR: reporting odds ratio; CI: confidence interval; rROR: relative ROR; IC: information component; CrI: credible interval; IC_Δ_: IC delta.

rROR (male vs female; reference = female).

IC_Δ_ (male vs female; reference = female).

**Table 8:** Comparison of FAERS and JADER Signals for levofloxacin-associated tendon disorders by sex and age.

	FAERS					JADER				
	*N*_11_	ROR (95% CI)	IC (95% CrI)	rROR (95%CI)	IC_Δ_ (95%CrI)	*N*_11_	ROR (95% CI)	IC (95% CrI)	rROR (95%CI)	IC_Δ_ (95%CrI)
ALL	6524	82.6 (80.3–85.1)	5.7 (5.7–5.8)			245	134.2 (112.5–160.2)	5.8 (5.6–6.0)		
Male	2863	87.2 (83.4–91.2)	5.8 (5.7–5.8)	1.09 (1.03–1.15)	0.05 (-0.002–0.10)	178	250.4 (196.1–319.6)	6.1 (5.8–6.3)	4.22 (2.89–6.17)	1.11 (0.86–1.29)
Female	3661	79.8 (76.7–82.9)	5.7 (5.7–5.7)			67	59.2 (44.3–79.1)	4.9 (4.5–5.2)		
Male										
< 20	16	66.4 (39.6–111.4)	4.4 (3.5–5.0)			0				
20–29	122	87.7 (70.8–108.6)	5.5 (5.2–5.7)			1	14.6 (1.6–131.4)	1.3 (-0.2–3.0)		
30–39	244	60.7 (52.3–70.6)	5.2 (5.0–5.4)			11	279.7 (77.5–1009.5)	4.0 (3.0–4.7)		
40–49	435	77.4 (68.9–86.9)	5.5 (5.3–5.6)			23	169.6 (86.4–333.1)	4.7 (4.0–5.2)		
50–59	489	75.2 (67.6–83.6)	5.6 (5.4–5.7)			6	43.3 (17.3–107.9)	3.2 (1.8–4.1)		
60–69	604	79.2 (71.9–87.2)	5.6 (5.5–5.7)			57	450.4 (273.5–741.8)	5.8 (5.4–6.1)		
70–79	580	110.5 (99.6–122.5)	5.9 (5.8–6.0)			63	478.8 (299.3–765.9)	5.9 (5.5–6.2)		
80–89	343	162.2 (139.7–188.2)	6.1 (5.9–6.2)			16	339.8 (132.3–872.7)	4.6 (3.7–5.1)		
≥ 90	30	233.7 (132.2–412.8)	5.1 (4.5–5.6)			1	360.8 (14.5–8976.1)	1.5 (-2.2–3.2)		
Female										
< 20	35	114.1 (79.1–164.7)	5.3 (4.7–5.7)			0				
20–29	104	92.4 (74.2–115.0)	5.7 (5.4–5.9)			0				
30–39	355	99.6 (87.8–113.0)	5.9 (5.7–6.0)			18	131.8 (62.9–276.1)	4.4 (3.6–4.9)		
40–49	652	81.3 (74.0–89.3)	5.6 (5.5–5.7)			2	13.3 (3.1–57.3)	1.9 (-0.6–3.3)		
50–59	976	87.8 (81.2–94.9)	5.7 (5.6–5.8)			26	114.1 (67.7–192.2)	4.8 (4.1–5.2)		
60–69	799	73.2 (67.4–79.6)	5.6 (5.4–5.6)			17	99.8 (54.4–182.9)	4.4 (3.6–5.0)		
70–79	487	67.6 (60.9–75.0)	5.5 (5.3–5.6)			2	11.6 (2.7–49.1)	1.8 (-0.7–3.2)		
80–89	229	83.5 (71.4–97.8)	5.6 (5.4–5.7)			2	46.3 (8.9–239.4)	2.1 (-0.4–3.5)		
≥ 90	24	68.7 (42.0–112.3)	4.5 (3.8–5.0)			0				

*N*_11_: the number of target-drug-induced target adverse event; ROR: reporting odds ratio; CI: confidence interval; rROR: relative ROR; IC: information component; CrI: credible interval; IC_Δ_: IC delta.

rROR (male vs female; reference = female).

IC_Δ_ (male vs female; reference = female).

**Table 9:** Levofloxacin-associated muscle, tendon, and ligament injuries and tendon disorders in JADER: Age (<70 vs ≥ 70) and sex stratified signals.

		*N*_11_	ROR (95% CI)	IC (95% CrI)	rROR (95%CI)	IC_Δ_ (95%CrI)
Muscle, tendon and ligament injuries						
< 70 years	ALL	163	40.7 (34.0–48.6)	4.9 (4.7–5.1)	0.51^a)^ (0.38–0.69)	–0.74^c)^ (−1.10– −0.48)
≥ 70 years	ALL	84	21.0 (16.2–25.4)	4.1 (3.8–4.4)		
< 70 years	Male	99	42.9 (34.0–54.0)	4.7 (4.4–4.9)	1.13^b)^ (0.78–1.63)	0.11^d)^ (−0.21–0.35)
	Female	64	37.7 (28.5–50.0)	4.5 (4.1–4.8)		
≥ 70 years	Male	79	35.6 (27.7–45.8)	4.5 (4.1–4.8)	12.21^b)^ (4.85–30.72)	3.18^d)^ (2.81–3.45)
	Female	5	2.9 (1.2–7.0)	1.2 (−0.2–2.2)		
Tendon disorders						
< 70 years	ALL	161	113.1 (91.5–139.8)	5.8 (5.7–6.0)	1.72 ^a)^ (1.16–2.55)	0.46 ^c)^ (0.09–0.72)
≥ 70 years	ALL	84	195.5 (140.8–271.3)	6.3 (6.1–6.5)		
< 70 years	Male	98	182.1 (134.2–247.2)	5.7 (5.4–6.0)	2.56^b)^ (1.66–3.96)	0.71^d)^ (0.38–0.95)
	Female	63	70.9 (52.1–96.6)	5.0 (4.6–5.3)		
≥ 70 years	Male	80	421.7 (277.3–641.0)	6.0 (5.6–6.3)	25.67^b)^ (8.37–78.72)	2.85^d)^ (2.48–3.11)
	Female	4	16.4 (5.8–46.4)	2.5 (0.7–3.6)		

*N*_11_: the number of target-drug-induced target adverse event; ROR: reporting odds ratio; CI: confidence interval; IC: information component; CrI: credible interval; IC_Δ_: IC delta.

a) rROR (≥ 70 years vs < 70 years; reference = < 70 years).

b) rROR (male vs female; reference = female).

c) IC_Δ_ (≥ 70 years vs < 70 years; reference = < 70 years).

d) IC_Δ_ (male vs female; reference = female).

### Comparison by route of administration in JADER

For levofloxacin, in the route-of-administration-stratified analyses, positive disproportionality signals for muscle, tendon, and ligament injuries and tendon disorders were detected irrespective of sex, and these signals were stronger with oral administration than with intravenous administration (Table 10).

**Table 10:** Levofloxacin-associated muscle, tendon, and ligament injuries and tendon disorders in JADER: route-of-administration (oral/intravenous) and sex stratified signals.

		*N*_11_	ROR (95% CI)	IC (95% CrI)
Muscle, tendon and ligament injuries				
ALL	Oral	222	34.7 (29.7–40.5)	4.5 (4.3–4.7)
	Intravenous	7	21.8 (10.2–46.5)	3.1 (1.8–4.0)
Male	Oral	161	42.5 (35.2–51.3)	4.7 (4.4–4.8)
	Intravenous	3	12.1 (3.8–38.3)	2.2 (0.1–3.4)
Female	Oral	61	23.8 (17.9–31.6)	4.0 (3.6–4.3)
	Intravenous	4	47.1 (16.9–130.8)	2.9 (1.1–4.0)
Tendon disorders				
ALL	Oral	223	194.7 (155.3–244.2)	5.8 (5.5–5.9)
	Intravenous	7	215.9 (93.5–478.4)	3.7 (2.4–4.6)
Male	Oral	163	311.4 (228.7–424.1)	5.9 (5.6–6.0)
	Intravenous	3	143.8 (41.5–497.8)	2.7 (0.6–3.9)
Female	Oral	60	96.2 (67.2–137.7)	5.1 (4.6–5.4)
	Intravenous	4	345.9 (109.7–1091.0)	3.1 (1.3–4.2)

*N*_11_: the number of target-drug-induced target adverse event; ROR: reporting odds ratio; CI: confidence interval; IC: information component; CrI: credible interval.

### Time-to-onset of levofloxacin-associated muscle, tendon, and ligament injuries and tendon disorders

**Fig 1:**
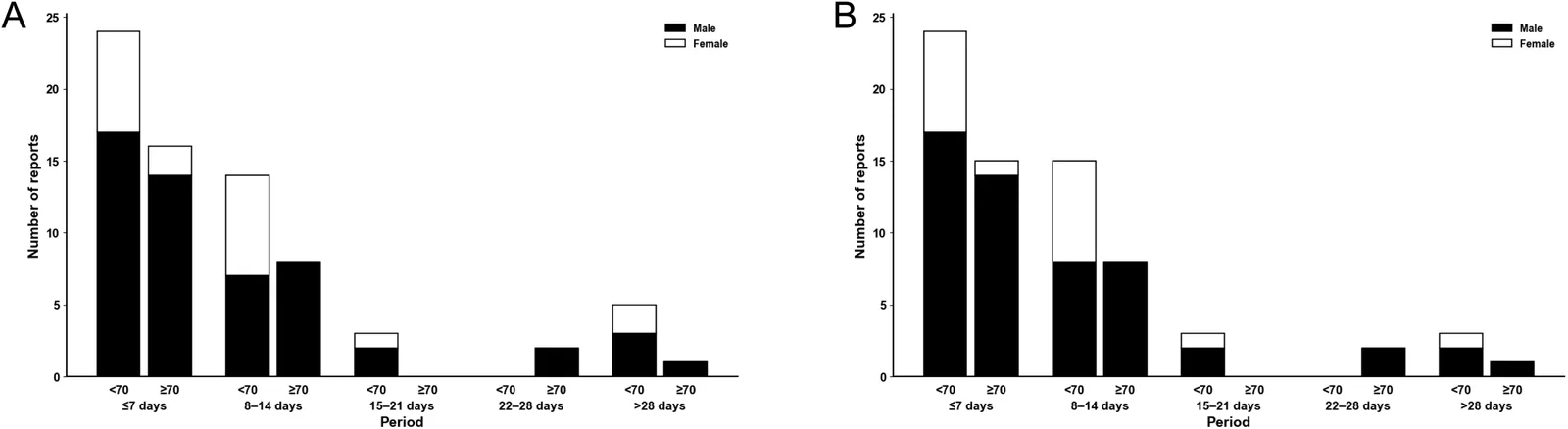
Onset time of levofloxacin-Induced muscle, tendon and ligament Injuries (A) and tendon disorders (B) in JADER.

For levofloxacin, the time-to-onset was available for 73 cases of muscle, tendon, and ligament injuries (54 males and 19 females) and 71 cases of tendon disorders (54 males and 17 females) in JADER. The time to onset of muscle, tendon, and ligament injuries and tendon disorders for levofloxacin is shown in Fig 1. Notably, most levofloxacin-associated muscle, tendon, and ligament injuries (median, 7 days; interquartile range [IQR], 4–11 days) and tendon disorders (median, 7 days; IQR, 4–11 days) occurred within 14 days. For levofloxacin, the time to onset did not differ by sex: muscle, tendon, and ligament injuries, 7 (4–10.75) days in males vs. 8 (3.5–11) days in females (P = 0.29); tendon disorders, 7 (4–10.75) days vs. 8 (3.5–11) days (P = 0.66). It also did not differ by age (≥ 70 vs < 70 years): muscle, tendon, and ligament injuries, 6 (4–10) days vs 7 (4–11.75) days (P = 0.66); tendon disorders, 6.5 (4–10.75) days vs 7 (4–11) days (P = 0.55).

## Discussion

Tendinitis and tendon rupture may occur in patients treated with fluoroquinolones. However, the onset characteristics of these adverse events have not yet been fully elucidated. This study was conducted using the FAERS and JADER databases to clarify the association between individual fluoroquinolones and tendon disorders and to assess differences by age, sex, and route of administration in the onset of muscle, tendon, and ligament injuries and tendon disorders associated with fluoroquinolones in the Japanese population, particularly levofloxacin.

In the overall FAERS, multiple fluoroquinolones showed positive disproportionality signals for muscle, tendon, and ligament injuries and for tendon disorders: ciprofloxacin (ROR_025_, 23.2 and 55.9; IC_025_, 4.2 and 5.3), levofloxacin (ROR_025_, 37.6 and 80.3; IC_025_, 4.8 and 5.7), moxifloxacin (ROR_025_, 8.4 and 15.4; IC_025_, 2.9 and 3.8), norfloxacin (ROR_025_, 9.7 and 17.0; IC_025_, 3.1 and 3.8), and ofloxacin (ROR_025_, 11.0 and 23.1; IC_025_, 3.3 and 4.3) (Tables 5 and 6). In the FAERS restricted to reports from Japan, signals were detected only for levofloxacin (ROR_025_, 6.7 and 6.7; IC_025_, 2.3 and 3.1) (Tables 5 and 6). This pattern aligns with the small contribution of Asian reports to the FAERS, as analyses of data on ciprofloxacin, levofloxacin, and moxifloxacin showed that Asian reports comprised 1.02% of suspected tendinitis and 1.28% of tendon rupture cases [11]. Accordingly, FAERS alone may be inadequate for analyses focused on Asia or Japan; therefore, JADER was used as a complementary data source. In JADER, although a weak positive disproportionality signal for tendon disorders was observed for ciprofloxacin (ROR_025_, 2.8; IC_025_, 0.3), strong signals for muscle, tendon, and ligament injuries and tendon disorders were most consistently observed for levofloxacin (ROR_025_, 26.6 and 112.5; IC_025_, 4.3 and 5.6). Findings from the FAERS reports restricted to Japan were consistent with those from JADER, identifying levofloxacin as the agent with the most consistent signal. These differences may reflect region-specific factors, including prescription patterns, drug utilization, concomitant medications, and potential genetic background. Because the preferred fluoroquinolone agents, indications, and routes of administration may differ across countries and regions, Japan-focused pharmacovigilance analyses are important for interpreting agent-specific safety signals in Japanese clinical practice. In the present analysis, levofloxacin showed the most consistent and robust disproportionality signals among the fluoroquinolones evaluated in FAERS-Japan and JADER. Consistent with this, a nationwide population-based cohort study in Taiwan reported that, after adjusting for seven fluoroquinolone agents, levofloxacin, norfloxacin, and pefloxacin were associated with a higher risk of tendon disorders [23]. These observations indicate that levofloxacin has the most consistent association with muscle, tendon, and ligament injuries and tendon disorders. In light of the Taiwanese cohort data, this raises the possibility of region-specific risk patterns within Asia.

In JADER, signals were absent for moxifloxacin and ofloxacin, and negligible for norfloxacin; ciprofloxacin showed a weak signal restricted to tendon disorders. Contrastingly, for levofloxacin, positive disproportionality signals for muscle, tendon, and ligament injuries and tendon disorders were detectable in both sexes but were consistently stronger in males than in females (Table 9). Despite these differences in signal strength by age and sex, time-to-onset distributions did not differ by sex or age in the JADER database; most levofloxacin-associated muscle, tendon, and ligament injuries and tendon disorders occurred within 14 days. Previous studies indicated that fluoroquinolone-associated tendon injuries increase with age, particularly among older adults. A meta-analysis on fluoroquinolones reported significantly elevated risks for Achilles tendinitis and rupture, remaining significant among patients ≥ 60 years. Complementary pharmacoepidemiologic studies showed a higher absolute risk in older adults [2,9]. Similarly, a nationwide cohort study in Taiwan observed an increased risk among those aged > 60 years [23]. Sex differences in fluoroquinolone-associated tendon injuries remain controversial. A large United Kingdom case-crossover study using the Health Improvement Network database suggested slightly stronger associations in females (Achilles tendinitis odds ratio, 5.0 vs 3.6), although the difference was not statistically significant [24]. Other FAERS-based analyses have reported a marginally higher proportion of female reports [11]. Conversely, several studies have reported male predominance of fluoroquinolone-associated tendinopathy [25,26]. While VigiBase noted a slight male predominance in reports within the ≥ 65 years subgroup across fluoroquinolones [10], our Japan-focused, age- and sex-stratified disproportionality analyses demonstrated a more pronounced sex difference at older ages, with males aged ≥ 70 years exhibiting significantly stronger levofloxacin disproportionality signals than females. Baseline epidemiology indicates that Achilles tendon rupture is more common in males, which may partly account for sex-specific patterns in pharmacovigilance datasets [27]. Nevertheless, our analyses suggest that older males, especially those aged ≥ 70 years, showed stronger disproportionality signals for levofloxacin-associated tendon disorders. Accordingly, these findings support increased clinical vigilance when prescribing levofloxacin to this population.

The mechanism of fluoroquinolone-induced tendon disorders remains unclear, but several mechanistic hypotheses have been proposed, including extracellular matrix degradation, impaired repair dynamics, oxidative stress, and mitochondrial dysfunction. Although mechanisms specific to levofloxacin have not been fully established, levofloxacin-associated tendon disorders may involve mechanisms proposed for fluoroquinolone-associated tendon toxicity. These processes may weaken tendon extracellular matrix integrity and reduce tendon resilience, particularly in older tendons. Older tendons exhibit decreased cellularity, impaired extracellular matrix remodeling, increased MMP-2 and MMP-9 activities, and reduced expression of tissue inhibitors of metalloproteinases, which potentially act via pathways overlapping those implicated in fluoroquinolone-associated injury [28,29]. Therefore, aging-related tendon vulnerability may have contributed to the stronger disproportionality signals in older patients.

Levofloxacin is commonly administered orally or intravenously. By analyzing FAERS, Shu *et al*. detected markedly stronger disproportionality signals for tendonitis and tendon rupture with oral versus intravenous fluoroquinolones, including levofloxacin [11]. In JADER, our levofloxacin-focused analysis showed a similar pattern of differential signal strength according to the patient subgroup and route. For muscle, tendon, and ligament injuries, signals were higher with oral administration than with intravenous injections (oral: *N*_11_, 222; ROR, 34.7; 95% CI, 29.7–40.5; IC, 4.5; 95% CrI, 4.3–4.7; intravenous: *N*_11_, 7; ROR, 21.8; 95% CI, 10.2–46.5; IC, 3.1; 95% CrI, 1.8–4.0). Similar disproportionality signals were also detected for tendon disorders (oral: *N*_11_, 223; ROR, 194.7; 95% CI, 155.3–244.2; IC, 5.8; 95% CrI, 5.5–5.9; intravenous: *N*_11_, 7; ROR, 215.9; 95% CI, 93.5–478.4; IC, 3.7; 95% CrI, 2.4–4.6). Furthermore, sex-stratified analyses showed that oral levofloxacin consistently yielded stronger disproportionality signals than intravenous levofloxacin. This pattern is consistent with the FAERS-based analyses findings for levofloxacin and indicates that the effect of the route of administration is replicated across spontaneous reporting systems. However, the reasons for the stronger oral signals remain uncertain. The route-specific difference may partly reflect differences in clinical setting and patient activity in addition to the route of administration. Oral levofloxacin may be more often used in outpatient settings, where patients may remain ambulatory and have greater physical activity-related tendon loading. In contrast, intravenous levofloxacin may be more often used in hospitalized or acutely ill patients who are relatively less active. Such tendon loading could act as a co-factor in tendon injury and may contribute to differences in reporting patterns by route of administration. However, this interpretation remains speculative because FAERS and JADER do not contain information on patient activity level, mobility, or tendon loading. Accordingly, the contribution of physical activity-related tendon loading could not be directly evaluated in this study. Differences in indication, disease severity, and care setting may also have influenced the observed route-specific signals.

This study had some limitations. The analysis relied on spontaneous reporting systems, such as FAERS and JADER. The data quality depends on voluntary reporting and the reporter’s expertise. Underreporting, selective or stimulated reporting, duplicate submissions, incomplete information, and misclassification can introduce bias in the estimates [19]. Disproportionality metrics, such as ROR, and IC, reflect reporting rather than incidence or risk and do not establish causality. These metrics cannot fully account for confounding by age, indication, dose, comorbidities, or concomitant medications, and residual confounding factors may remain [30]. Among concomitant medications, systemic corticosteroids are particularly relevant because systemic corticosteroid therapy is a recognized risk factor for tendon injury and has been associated with a high risk of fluoroquinolone-associated tendon disorders [9,24]. In the present analysis, concomitant corticosteroid therapy and other concomitant medications were not used as exclusion criteria, and their timing, dose, and duration could not be reliably determined from FAERS or JADER. Therefore, residual confounding by corticosteroids and other concomitant medications may have influenced the observed disproportionality signals.

Renal function represents another important source of potential residual confounding, particularly in the interpretation of levofloxacin-associated signals, because impaired renal function may increase systemic exposure. However, FAERS and JADER do not contain structured laboratory data, such as serum creatinine or estimated glomerular filtration rate, and renal impairment-related Preferred Terms recorded as medical history, comorbid conditions, or adverse events do not necessarily indicate baseline renal function at the time of fluoroquinolone initiation. In addition, clinicians may avoid fluoroquinolones or adjust their doses in patients with renal impairment. Therefore, the reporting patterns in patients with renal impairment may not reflect the association under comparable dosing or exposure conditions. Accordingly, we could not reliably stratify reports by renal function or evaluate dose-adjusted exposure, and the present levofloxacin findings should be interpreted with caution in light of possible residual renal confounding.

Time-to-onset analyses based on spontaneous reporting systems should also be interpreted cautiously [31]. In FAERS and JADER, onset dates and treatment initiation dates may be incomplete, imprecise, or inconsistently recorded. In addition, tendon-related symptoms may develop gradually, and the reported onset date may reflect the time of clinical recognition rather than the true biological onset. Because only reports with sufficient temporal information were included in the time-to-onset analysis, selection bias may also have influenced the observed onset patterns. Therefore, the present time-to-onset findings should be regarded as descriptive and hypothesis-generating rather than precise estimates of onset timing.

Finally, exposure denominators are not available, which prevents the estimation of absolute risks and the comparison of incidence across fluoroquinolones [19]. Therefore, the findings should be viewed as hypothesis-generating and should be confirmed in population-based studies with known denominators and rigorous confounding control.

In this study, we analyzed FAERS data to characterize Japan-specific disproportionality for muscle, tendon, and ligament injuries and tendon disorders; however, this approach was insufficient. Therefore, we analyzed JADER data to obtain Japan-focused **signal estimates**. In JADER, only levofloxacin showed positive signals for both adverse effects. Signals were detectable in both sexes but were consistently higher in males, particularly in those aged ≥ 70 years. In such cases, levofloxacin should be used with caution, considering the potential for tendon-associated adverse events. Most levofloxacin-associated tendon disorders occurred within 14 days after treatment initiation. These findings support close monitoring for symptoms suggestive of tendon injury during the first 2 weeks after levofloxacin initiation, particularly in older male patients. Further population-based studies are needed to confirm these findings and to evaluate the contribution of physical activity-related tendon loading to tendon injury in this demographic.

## Supporting information

S1 Table Drug identification terms used for fluoroquinolones. (DOCX)

S2 Table Preferred Terms included in the target HLTs. (DOCX)
