# Supplementary material for: Levofloxacin-associated tendon disorders: Sex-, age-, and route-specific disproportionality analysis using FAERS and JADER
Source: PLoS One. 2026 Sep 11;21(9):e0358427. doi: 10.1371/journal.pone.0358427 (PMC13568500; doi:10.1371/journal.pone.0358427)
Supplement: Supplementary file 1 [file pone.0358427.s001.docx]

S1 Table Drug identification terms used for fluoroquinolones.

| Fluoroquinolone | Drug identification terms |
| --- | --- |
| Ciprofloxacin | ciprofloxacin, ciprofloxacin hydrochloride, ciprofloxacin lactate |
| Levofloxacin | levofloxacin, levofloxacin mesilate, levofloxacin hydrochloride, levofloxacin lactate |
| Moxifloxacin | moxifloxacin, moxifloxacin hydrochloride |
| Norfloxacin | norfloxacin, norfloxacin hydrochloride |
| Ofloxacin | ofloxacin, ofloxacin hydrochloride |
