# Supplementary material for: Levofloxacin-associated tendon disorders: Sex-, age-, and route-specific disproportionality analysis using FAERS and JADER
Source: PLoS One. 2026 Sep 11;21(9):e0358427. doi: 10.1371/journal.pone.0358427 (PMC13568500; doi:10.1371/journal.pone.0358427)
Supplement: Supplementary file 2 [file pone.0358427.s002.docx]

S2 Table Preferred Terms included in the target HLTs.

| HLT | Preferred Terms |
| --- | --- |
| Muscle, tendon and ligament injuries | Epicondylitis, Ligament sprain, Mallet finger, Muscle haemorrhage, Muscle injury, Muscle rupture, Repetitive strain injury, Rotator cuff syndrome, Tendon disorder, Tendon injury, Tendon rupture, Tendonitis, Muscle strain, Sinus tarsi syndrome, Haematoma muscle, Iliotibial band syndrome, Ligament injury, Iliolumbar syndrome, Ligament rupture, Joint capsule rupture, Muscle hernia, Muscle contusion, Post-traumatic neck syndrome, Muscle oedema, Fascial rupture, Pellegrini Stieda disease, Aponeurosis contusion, Tensor fasciae latae syndrome, Tendon dislocation, Posterior tibial tendon dysfunction, Sacroiliac joint dysfunction, Rotator cuff injury of hip, Rotator cuff tear arthropathy, Gluteus medius syndrome |
| Tendon disorders | Enthesopathy, Epicondylitis, Tendinous contracture, Tendon disorder, Tendon injury, Tendon rupture, Tendonitis, Tenosynovitis, Trigger finger, Tenosynovitis stenosans, Tendon calcification, Tuberculous tenosynovitis, Tendon pain, Tendon necrosis, Tendon laxity, Tendon discomfort, Tendon sheath disorder, Tendon dislocation, Enthesophyte, Intersection syndrome, Posterior tibial tendon dysfunction, Tendon sheath effusion, Gluteus medius syndrome, Trigger toe |
